# The N-terminal signature motif on the transporter MCT1 is critical for CD147-mediated trafficking

**DOI:** 10.1016/j.jbc.2024.107333

**Published:** 2024-05-22

**Authors:** Devin J. Seka, Annika K. Schulz, Tarjani M. Thaker, Thomas M. Tomasiak

**Affiliations:** Department of Chemistry and Biochemistry, University of Arizona, Tucson, Arizona, USA

**Keywords:** monocarboxylate transporters, lactate, pyruvate, membrane complexes, thermostability, trafficking, subcellular localization

## Abstract

The human Solute Carrier (SLC) family member, monocarboxylate transporter 1 (MCT1), transports lactic and pyruvic acid across biological membranes to regulate cellular pH and metabolism. Proper trafficking of MCT1 from the endoplasmic reticulum to the plasma membrane hinges on its interactions with the membrane-bound chaperone protein, CD147. Here, using AlphaFold2 modeling and copurification, we show how a conserved signature motif located in the flexible N-terminus of MCT1 is a crucial region of interaction between MCT1 and the C-terminus of CD147. Mutations to this motif—namely, the thymic cancer linked G19C and the highly conserved W20A—destabilize the MCT1-CD147 complex and lead to a loss of proper membrane localization and cellular substrate flux. Notably, the monomeric stability of MCT1 remains unaffected in mutants, thus supporting the role of CD147 in mediating the trafficking of the heterocomplex. Using the auxiliary chaperone, GP70, we demonstrated that W20A-MCT1 can be trafficked to the plasma membrane, while G19C-MCT1 remains internalized. Overall, our findings underscore the critical role of the MCT1 transmembrane one signature motif for engaging CD147 and identify altered chaperone binding mechanisms between the CD147 and GP70 glycoprotein chaperones.

Monocarboxylate transporter 1 (MCT1) is a pivotal member of the SLC16 family, which comprises 14 distinct isoforms with crucial roles in cellular metabolism and hormone signaling (1, 2). Notably, isoforms 1 through 4 are lactic acid and pyruvic acid transporters, with MCT4 exporting lactic acid in glycolytic cells and MCT1/2 importing lactic acid in oxidative cells (3). These synergistic functions occur in the reverse Warburg effect, a hallmark of cancers (4). Beyond its role in malignancies, disruptions to the MCT1-CD147 complex have been implicated in a spectrum of diseases, including the progression of diabetes (5) and recurrent ketoacidosis (6). The loss of MCT1 expression and activity emerges as a critical factor leading to reduced uptake of ketone bodies, lactic acid, and pyruvic acid, resulting in a consequential decrease in blood pH and subsequent metabolic and neurological repercussions (7).

MCT1-4 are heteromeric solute carriers dependent on chaperones for quality control and the regulation of expression levels and cellular trafficking (2). MCT1 is tightly regulated by the co-expressed transmembrane chaperones, CD147 (Basigin/EMMPRIN) or GP70 (Embigin) (8). CD147, the primary MCT1 chaperone, is widely expressed throughout the body (9) and is required for MCT1 trafficking to the plasma membrane. In contrast, GP70 is differentially expressed in the outer segment of photoreceptors in the retina (10), neurons (11), and during embryonic development (12). Both chaperones have multiple, extracellular Ig domains and a disordered, intracellular C-terminal domain (CTD) connected by a single transmembrane helix (13). Using the sequence numbering for isoform two of MCT1’s preferred chaperone, CD147, the cytosolic tail is approximately residues 230 to 269. Although the specific interactions that govern chaperone preference are not well defined, the tight MCT1-chaperone association is essential for transporter localization and, thus, activity (8, 14, 15, 16).

The MCT family contains the highly conserved consensus sequence, [D/E]G[G/S][W/F][G/A]W, situated where the disordered N-terminal tail transitions into the beginning of transmembrane helix 1 (TM1) (Fig. S1). In MCT1, this signature motif is specifically 15-DGGWGW-20. Previous research analyzing one residue from the motif, D15, found that the MCT1 D15N variant displayed reduced surface expression and lower cellular substrate flux in oocytes (17). Although the functions of W18 and W20 in MCT1 have not been studied, previous research determined that they facilitate homodimerization and cooperative transport in MCT2 (18).

The cryo-EM structures of MCT1-CD147 and MCT1-GP70 (19, 20) reveal the transmembrane and extracellular Ig domains of the chaperones, with only partial resolution of the cytosolic termini of the complexes (Fig. S2). The structures reveal a more extensive Ig domain interaction in MCT1-GP70 compared to MCT1-CD147, but largely similar transmembrane interactions. The association of CD147 with MCT1 is primarily driven by a series of transmembrane hydrophobic interactions and one critical salt bridge between E218 on CD147 and N187 on TM6 of MCT1 (20). Crucially, the N-terminal ectodomain of CD147 does not significantly interact with MCT1 (20). Although GP70 forms analogous transmembrane interactions with MCT1, it also forms several contacts through the Ig domains, including an additional extracellular salt bridge pairing R175 on MCT1 to D174 on GP70 and extensive hydrophobic interactions (19). The signature motif is only partially resolved in these structures, but considering its proximity to where CD147 dimerizes with MCT1, alterations likely disrupt the stability of the MCT1-CD147 complex.

Here, we show the molecular basis for CD147-dependent regulation of MCT1 localization and stability, which has a direct effect on cellular substrate flux. Using orthogonal and complementary techniques in bioinformatics, biochemistry, and confocal microscopy, we mutated key residues along the MCT1 N-terminal signature motif to determine its role in CD147-mediated trafficking functions. We also show how an MCT1 mutation present in a thymic cancer case (21) reduces MCT1 function by interfering with the chaperone interaction. Furthermore, we distinguish structural and functional differences in the association of MCT1-CD147 and MCT1-GP70 in the unmodeled cytosolic interface of the complexes.

## Results

### The N-terminus and TM1 of MCT1 are predicted to interact with CD147

**Figure 1:**
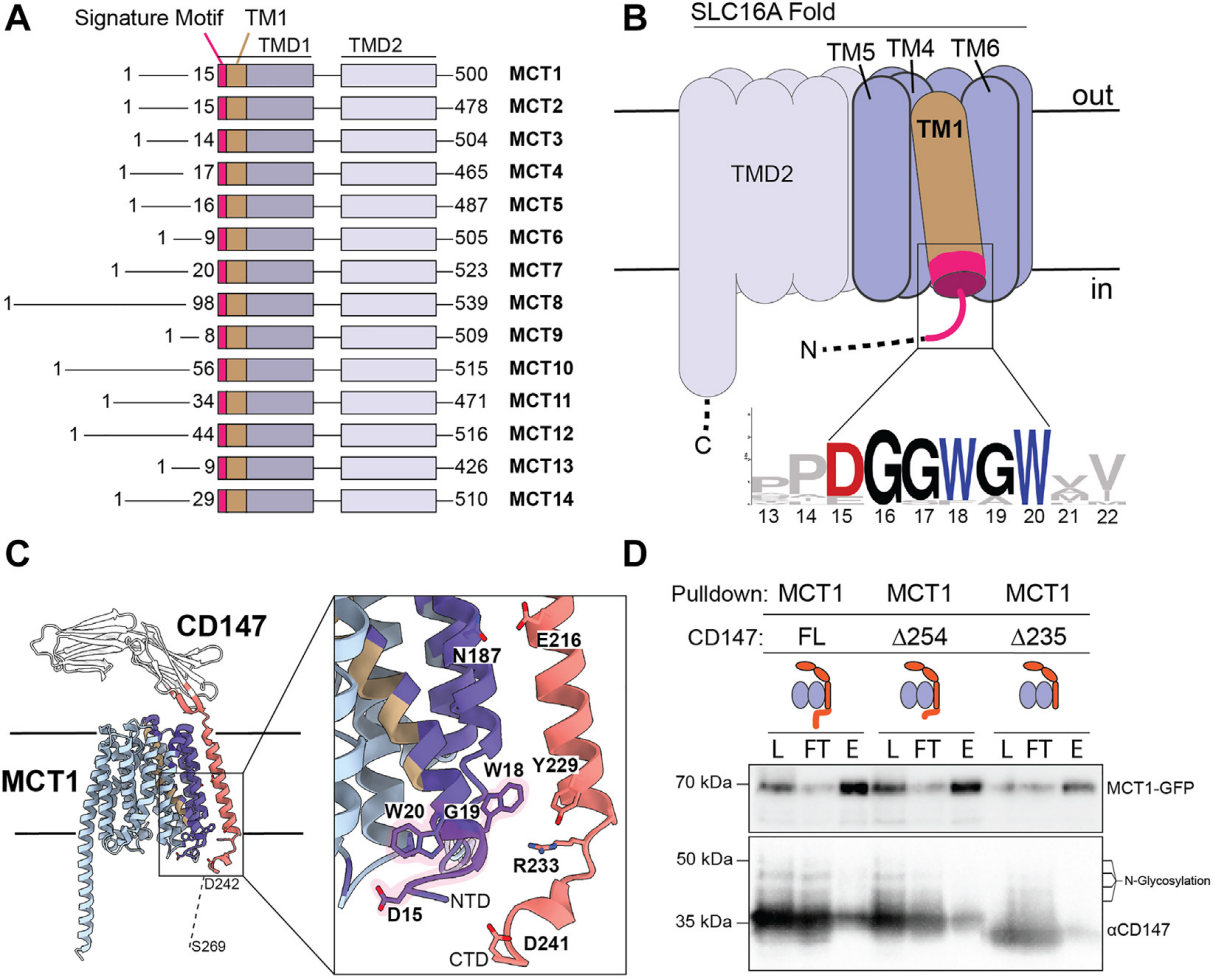
**Predicted interface of MCT1-CD147 includes the signature motif on TM1 of MCT1 and the C-terminus of CD147.***A*, alignment of domains and motifs in the MCT family. *B*, MCT family topology with the signature motif at transmembrane helix one depicted with a WebLogo (43) from sequences of the entire human MCT family. *C*, Alphafold2-based FoldDock model of MCT1-CD147 with predicted interacting residues of MCT1 in cartoon (*purple*) and CD147 in cartoon (*orange*). The signature motif from the MCT family is highlighted in *light pink*. *D*, flag-tag pulldown on the MCT1 subunit with CD147 full-length (FL), truncated C-terminal 16 amino acids (Δ254), and truncated C-terminal 35 amino acids (Δ235); load (L), flow through (FT), and elution (E) samples shown for each.

According to recent cryo-EM structures, the MCT1-CD147 complex is mainly stabilized by interactions through the transmembrane helix of CD147 to TM6 (19, 20). The incompletely resolved N- and C-termini of MCT1 and CD147, respectively, include part of the signature motif. Considering its likely proximity to the CD147 C-terminus, we hypothesize that the intermolecular interaction may also involve the MCT1 N-terminus (Fig. 1, *A* and *B*).

To gain insight into a putative mechanism for CD147 engagement of MCT1, we utilized AlphaFold2-based protein interaction predictions by FoldDock, which performs simultaneous folding and docking calculations (22), to evaluate the missing region between TM1 of MCT1 and the CD147 C-terminal tail (Fig. 1, *C* and *D*). Predicted interacting residues identified by FoldDock confirm known interactions from MCT1 TM6, including a critical N187 residue. The FoldDock model also predicts new interactions between D15, W18, G19, and W20 of the signature motif to residues 229 through 241 of CD147 (Fig. 1*C*). These results suggest that the N-terminal motif on MCT proteins may be important for oligomerization in the family.

### The cytosolic tail of CD147 is necessary for complex association

Truncations to the cytosolic tail of CD147 were created to examine the contribution of the CD147 CTD to heterodimer formation. Deleting the terminal 16 residues of CD147 (Δ254–269) did not impact MCT1-CD147 copurification, suggesting these residues are non-essential in the protein-protein interaction. However, truncation of the terminal 35 residues of CD147 (Δ235–269) resulted in the loss of complex copurification, implying that this CTD region is essential for complex stability (Figs. 1*D* and S1*B*). The five charged amino acids (230-EKRRK-235) positioned C-terminally to the transmembrane helix were present in the truncated CD147-∆235 variant and are likely responsible for proper membrane insertion of CD147 (23, 24). Overall, the loss in stability arising from CD147 tail truncations suggests that an interaction between the CD147 C-terminus and MCT1 is important for complex association and trafficking.

### Mutations to the signature motif decrease membrane expression of MCT1 and CD147

**Figure 2:**
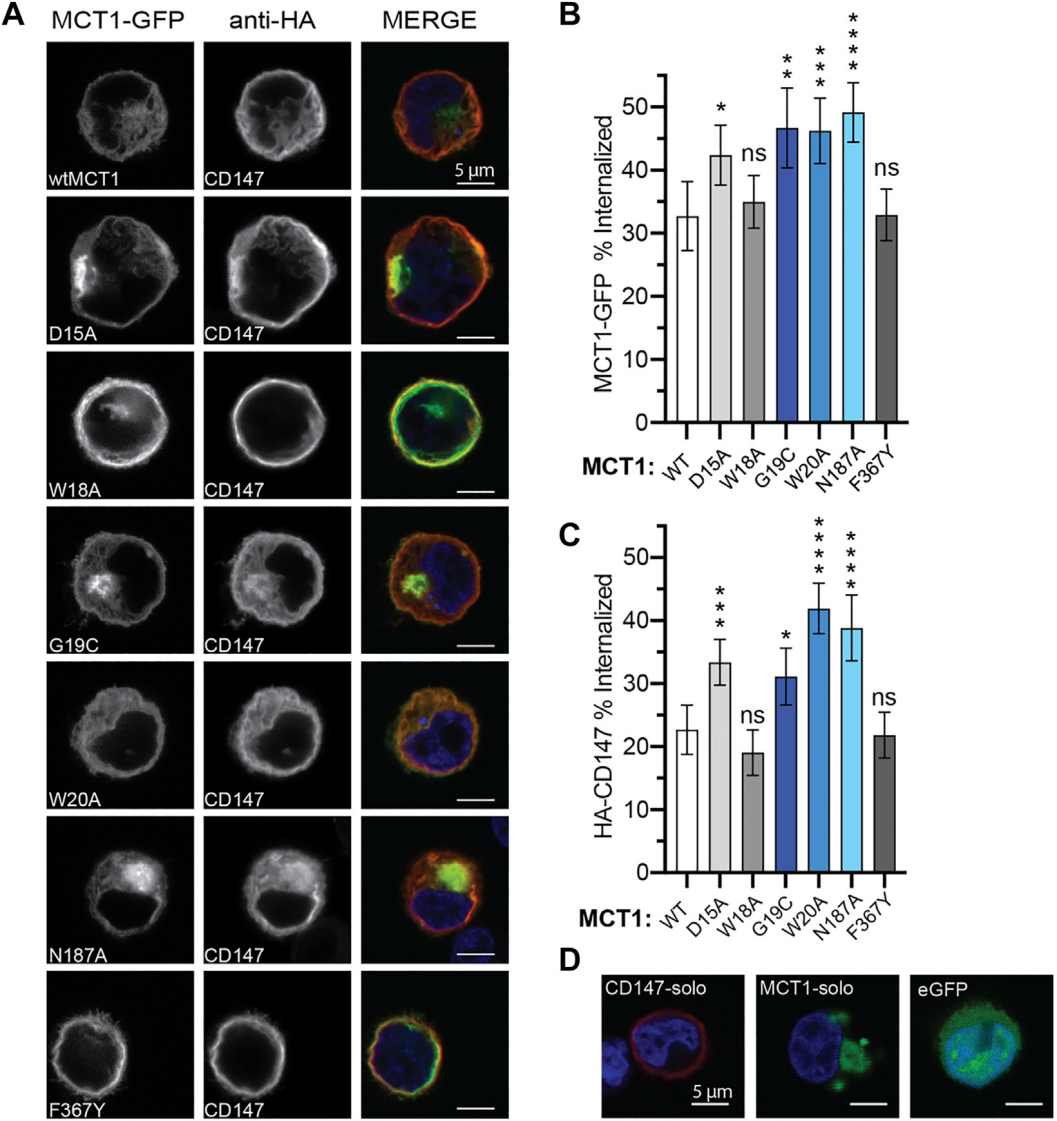
**MCT1 is dependent on CD147 for trafficking to the plasma membrane, and mutant MCT1 variants have reduced surface expression.***A*, immunofluorescent confocal microscopy on transfected Expi293F cells expressing MCT1 variants (*green*) and CD147 (*red*). *B*, quantification of localization of MCT1-GFP signal below 70% of the cell radius as a percent of the total signal. *C*, internalization of HA-CD147 subunit in the presence of MCT1 variants. *D*, CD147 alone properly localizes to the plasma membrane, and the solo MCT1 transfection results in internalized transporter. Monomeric eGFP is diffused through the cell. Data analyzed with a one-way ANOVA and Dunnett's test, mean ± 95% CI, at least 45 cells total per biological triplicate with at least 12 cells per replicate, (ns) = *p* > 0.05, ∗*p* ≤ 0.05, ∗∗*p* ≤ 0.01, ∗∗∗*p* ≤ 0.001, ∗∗∗∗*p* ≤ 0.0001.

To measure the impact of MCT1 signature motif mutations, we imaged transfected Expi293F cells with confocal microscopy to assess the plasma membrane trafficking of wild-type and mutant MCT1-GFP and HA-CD147 (Fig. 2*A*). Cells expressing CD147 and one of several MCT1 variants were analyzed for surface expression (Fig. 2, *A*–*C*). These include wild-type MCT1, mutant variants with missense mutations to the N-terminal signature motif (D15A, W18A, G19C, and W20A-MCT1), and two controls: a known complex disrupter, N187A-MCT1 (15, 16), and an active site mutant, F367Y-MCT1 (25). Using manually added cell perimeters in the ImageJ multi-clock scan plugin (26), we quantitatively evaluated the effects of these mutations on cell surface presentation (Figs. 2, *B* and *C* and S3).

**Figure 3:**
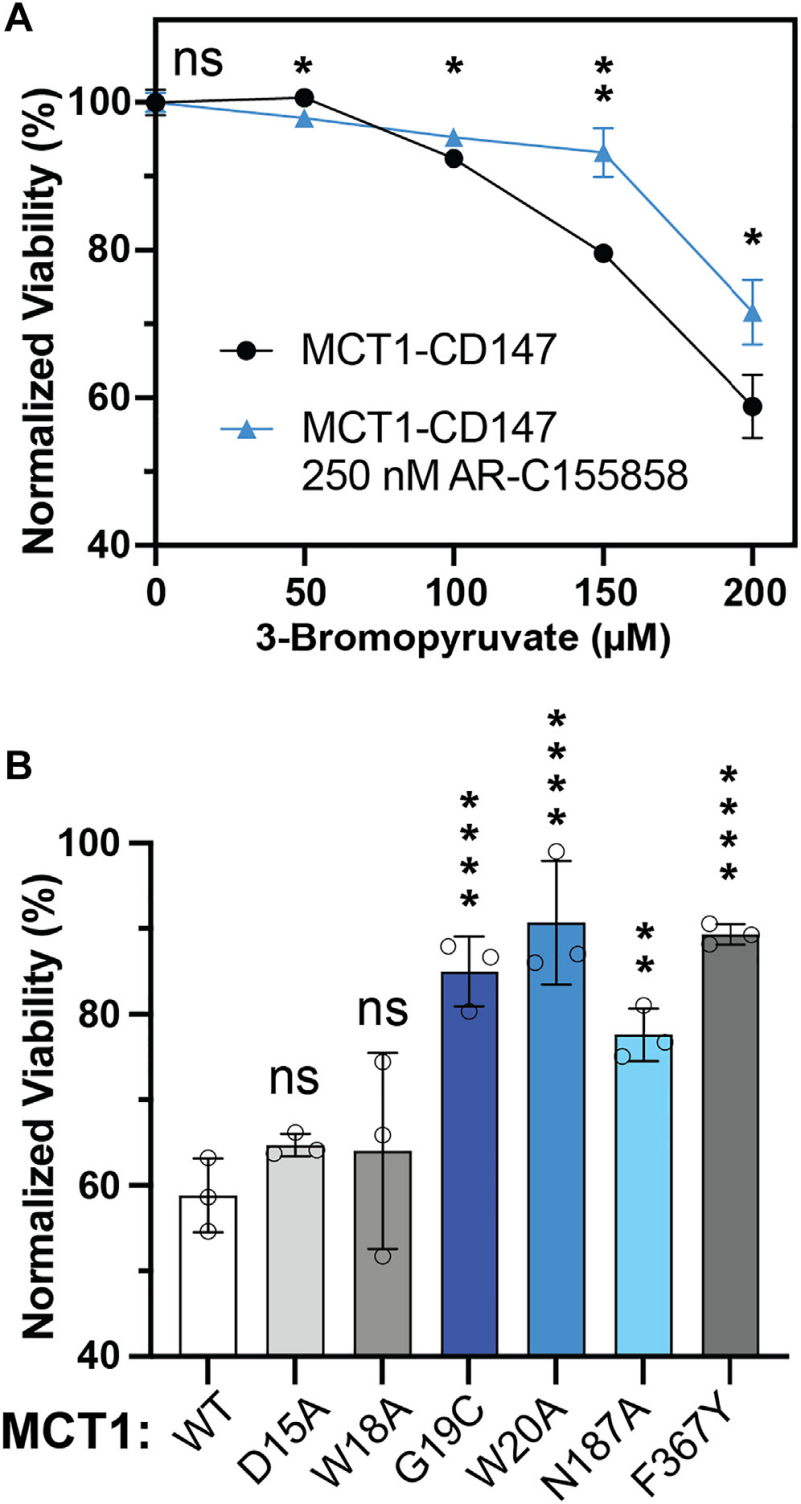
**Cytotoxic uptake assay of 3-bromopyruvate in cells expressing MCT1-CD147 variants.***A*, viability curve for the wild-type complex uptake of 3-bromopyruvate and inhibitor response curve. Data shown as mean ± SD, biological triplicate, with an unpaired *t* test, (ns) = *p* > 0.05, ∗*p* ≤ 0.05, ∗∗*p* ≤ 0.01. *B*, endpoint viability of MCT1 mutants co-expressed with CD147 in the presence of 200 μM of 3-bromopyruvate. Data analyzed with a one-way ANOVA and Dunnett's test, mean ± SD, biological triplicate, (ns) = *p* > 0.05, ∗*p* ≤ 0.05, ∗∗*p* ≤ 0.01, ∗∗∗*p* ≤ 0.001, ∗∗∗∗*p* ≤ 0.0001.

Wild-type MCT1-CD147 properly localized to the plasma membrane as expected, as did the transport-deficient (25) F367Y-MCT1 mutant. Conversely, mutation of residues suspected to interact with CD147, namely G19 and W20, directly resulted in reduced surface expression of both subunits (Fig. 3, *B* and *C*). The impact of these mutations is commensurate with that of the transmembrane mutant, N187A-MCT1, which was significantly intracellularly retained. The D15A-MCT1 mutant was partially internalized, consistent with previous literature reports (17). W18A-MCT1 was still present at the membrane and not significantly internalized compared to wild-type MCT1-CD147. A solo transfection of both constructs was also performed to demonstrate MCT1 dependence on CD147 for cellular localization. In this experiment, MCT1-solo was heavily internalized, whereas CD147 could reach the plasma membrane without its client (Fig. 2*D*).

### MCT1 TM1 mutations result in decreased cellular substrate flux

MCT1 transports a host of monocarboxylate substrates, including lactate, pyruvate, butyrate, and ketone bodies (17, 27). The transporter complex is also capable of importing the anticancer drug, 3-bromopyruvate (3-BP), which is toxic to cells (28, 29). Here, 3-BP toxicity was used as a proxy for MCT1 transport activity in Expi293F cells. Cells expressing MCT1-CD147 were sensitive to 3-BP treatment and exhibited a dose-dependent decrease in cellular viability when exposed to the substrate for 24 h (Fig. 3*A*). The loss in viability was rescued with the MCT1/2 inhibitor, AR-C155858, at a 250 nM concentration, which is approximately 100 times its K_i_ in rat red blood cells and in *Xenopus laevis* oocytes (30).

The trafficking mutants were also tested for sensitivity to 3-BP treatment. Cells expressing the destabilizing mutants, G19C, W20A, and N187A, showed increased viability in the presence of 3-BP compared to the wild-type complex (Fig. 3*B*). Similarly, cells expressing the active site mutant, F367Y-MCT1, exhibited resistance to treatment with 3-BP. The mutants, D15A and W18A-MCT1, were sensitive to 3-BP treatment, displaying a similar loss of cellular viability compared to the wild-type complex. Increased resistance indicates a lack of 3-BP transport activity, resulting from either the absence of transporters at the cell surface or the reduced activity of properly localized transporters.

### Mutations to residues on the N-terminal motif of MCT1 decrease complex thermostability

**Figure 4:**
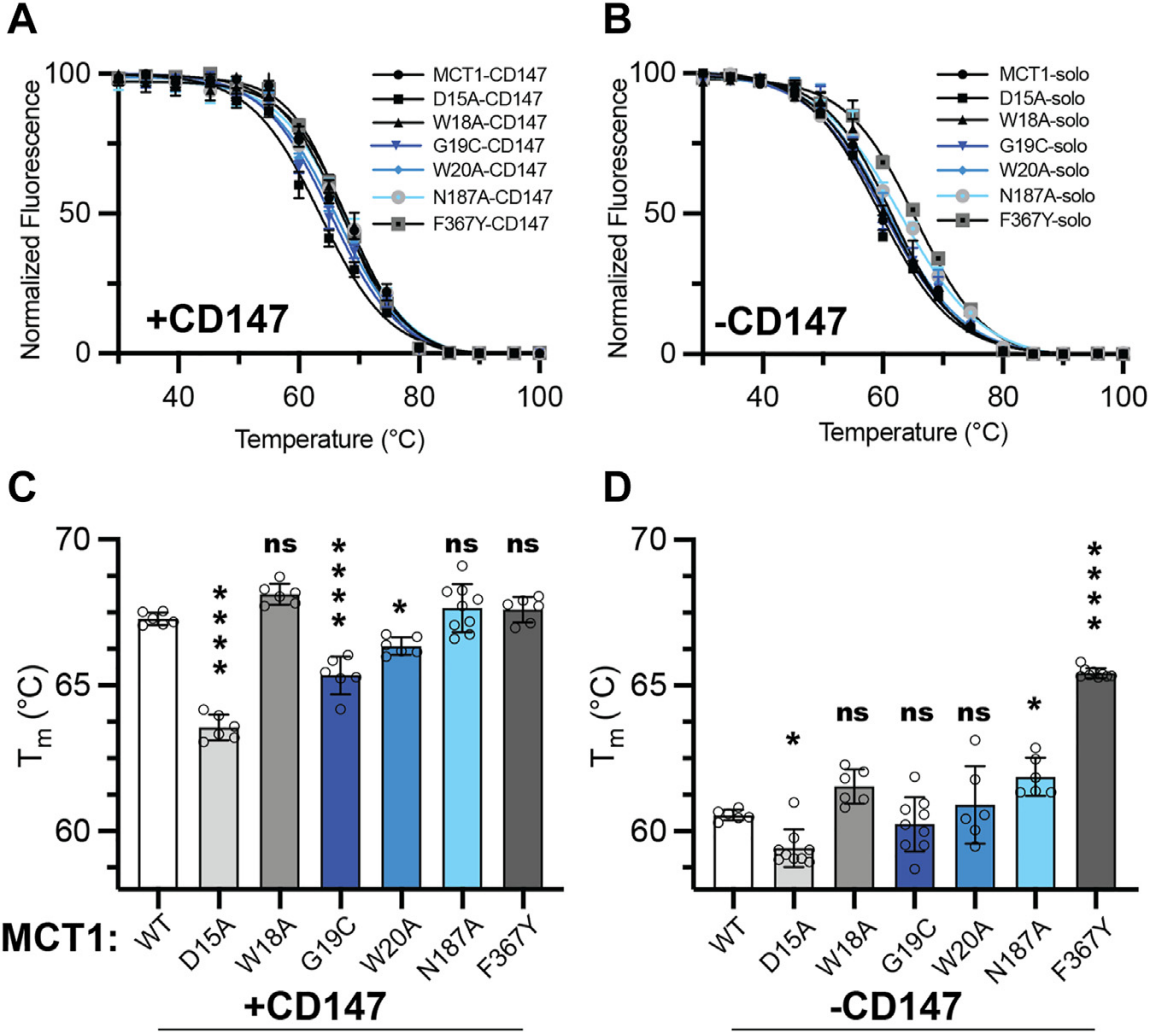
**Mutations to MCT1 N-terminal motif destabilize the MCT1-CD147 complex.***A*, GFP-based crude membrane thermal shift assay of MCT1 mutants with the co-expression of recombinant CD147. *B*, thermal shift curves of MCT1 solo expression. *C*, bar graph representation of MCT1 thermal aggregation temperatures in the presence of CD147. *D*, thermal aggregation temperatures for MCT1 mutants in the absence of CD147. Data anlyzed with a one-way ANOVA and Dunnett's test, mean ± SD, n ≥ 6 biological duplicate, (ns) = *p* > 0.05, ∗*p* ≤ 0.05, ∗∗*p* ≤ 0.01, ∗∗∗*p* ≤ 0.001, ∗∗∗∗*p* ≤ 0.0001.

The impact of CD147 on MCT1 stability or folding in a biochemically reconstituted system has not been explored before. We assessed the thermal aggregation of MCT1, both in the presence and absence of CD147, utilizing a crude membrane GFP thermal shift assay (31) (Fig. 4, *A* and *B*). The co-expression of CD147 with MCT1 increased the thermal stability of MCT1-GFP by approximately 7 °C compared to MCT1-GFP solo expression (Fig. 4 and Table 1).

**Table 1:** Thermal aggregation temperatures (°C) of MCT1-GFP variants in the presence and absence of CD147

MCT1	WT	D15A	W18A	G19C	W20A	N187A	F367Y
+CD147	67.28 ± 0.21	63.55 ± 0.44	68.12 ± 0.36	65.33 ± 0.65	66.36 ± 0.30	67.64 ± 0.82	67.59 ± 0.44
−CD147	60.54 ± 0.18	59.41 ± 0.64	61.53 ± 0.59	60.23 ± 0.93	60.90 ± 1.33	61.87 ± 0.66	65.24 ± 0.17

Mean ± SD.

Intriguingly, the N187A mutant, known to disrupt complex formation between MCT1 and its chaperones, did not decrease MCT1 thermostability in the presence of CD147. This is likely attributed to N187 being a polar residue embedded in the hydrophobic lipid bilayer, where its mutation to a small nonpolar alanine was stabilizing. The thermal aggregation of W18A-MCT1 and of the transport deficient F367Y-MCT1 were not significantly impacted (Fig. 4). However, the G19C and W20A mutant transporters were significantly destabilized compared to wild-type MCT1-CD147. Although unclear in mechanism, these results demonstrate that G19 and W20 are essential residues for complex stability. Co-expression of both subunits, MCT1 and CD147, was confirmed by Western blot analysis for all mutants and indicated that loss of MCT1 stability was likely directly related to the biomolecular interaction (Fig. S4).

To test if the point mutations inherently destabilize MCT1, we performed a thermostability assay on the solo MCT1 variants. There was no significant difference between solo MCT1 and W18A, G19C, and W20A melting points, suggesting the N-terminal motif does not substantially affect intramolecular stability. The D15A mutation reduced the monomeric melting point, but only by a small (∼1 °C) amount. Both the control N187A and F367Y mutants stabilized MCT1 to various degrees (Fig. 4*D*). This further implicates the signature motif residues, G19 and W20, in facilitating the higher-order oligomeric structure.

### GP70 can traffic W20A but not G19C-MCT1 to the cell surface

**Figure 5:**
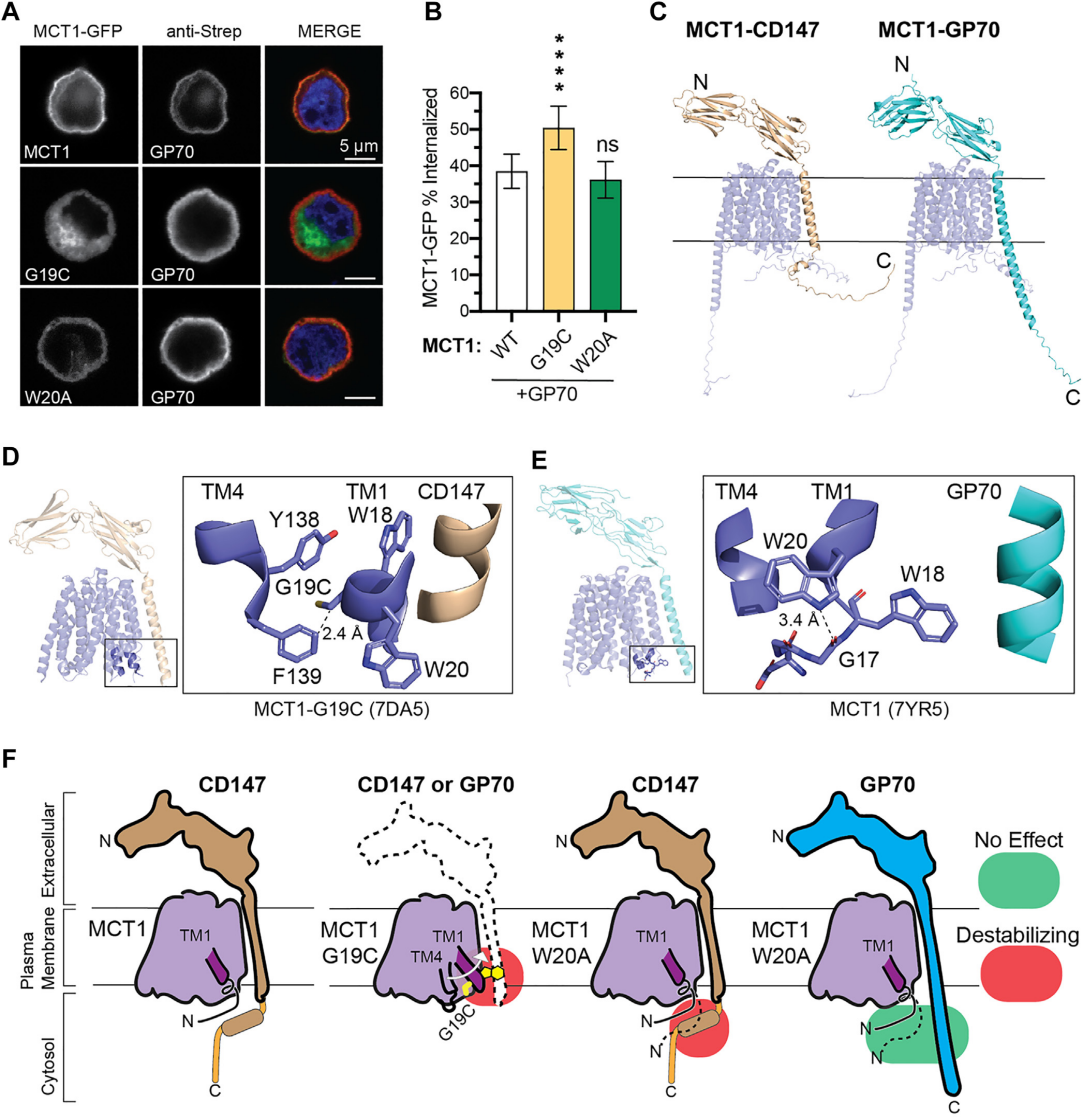
**Auxiliary chaperone GP70 can traffic W20A-MCT1 but not G19C-MCT1 due to CTD conformations.***A*, representative micrographs of MCT1 variants (*green*) with GP70 co-expressed (*red*). *B*, quantification of MCT1-GFP signal present at 0 to 70% of cell radius. *C*, alphafold2.3-multimer models of CD147 or GP70 in complex with wild-type MCT1. *D*, MCT1-CD147 (7DA5) with cystine substituted for glycine19, and notable clashes with TM helix four of MCT1. *E*, the W20 residue (7YR5) stabilizes N-terminal conformations by interacting with the G17 peptide backbone. *F*, wild-type protein with properly folded termini. G19C mutation induces TM1 movement, clashing with both CD147 and GP70 transmembrane helices. W20A mutation unwinds the TM1 helix clashing with the termini interaction of CD147 but has no effect on GP70-MCT1 dimerization. Data analyzed with a one-way ANOVA and Dunnett's test, mean ± 95% CI, at least 45 cells total per biological triplicate with at least 12 cells per replicate, (ns) = *p* > 0.05, ∗*p* ≤ 0.05, ∗∗*p* ≤ 0.01, ∗∗∗*p* ≤ 0.001, ∗∗∗∗*p* ≤ 0.0001.

To evaluate the contribution of the signature motif to chaperone specificity between CD147 or GP70, we tested the effect of the CD147-trafficking deficient mutants, G19C-MCT1 and W20A-MCT1, on GP70-mediated trafficking. As a control, we showed GP70 trafficked wild-type MCT1 to the plasma membrane (Fig. 5*A*). When co-expressed with GP70, G19C-MCT1 remained internalized to a similar extent as when co-expressed with CD147. However, significant changes were observed with W20A-MCT1, which showed almost no loss of trafficking compared to wildtype MCT1-GP70 (Fig. 5, *A* and *B*). Again, MCT1 solo fails to properly localize to the cell surface without a trafficking chaperone co-expressed (Fig. 2*D*).

To develop a possible mechanism for chaperone specificity involving the signature motif, we utilized Alphafold2 to generate structures of full-length MCT1-GP70 and MCT1-CD147. The models revealed key differences in the CTD's of each chaperone. The CD147 CTD is near the cytosolic interface of the MCT1 N-terminus, while the GP70 CTD is helical and projects away from this region of MCT1 (Fig. 5*C*). Furthermore, as seen in the cryo-EM structures (19, 20), the residues of the signature motif span the transition from disordered (residues ∼15–17) to helical (residues 18–20) regions. Therefore, G19C and W20A may perturb other nearby elements and their interactions with chaperones. In particular, the W20 residue hydrogen bonds to the G17 peptide backbone in MCT1 TM1 (Fig. 5*E*) and may destabilize the N-terminus when missing (Fig. 5*F*). This would impact binding to CD147 but not GP70 (Fig. 5, *C* and *F*). G19C, on the other hand, may cause a displacement of TM1 by inducing a steric clash between the cysteine side chain and TM4 (Fig. 5, *D* and *F*). In summary, the MCT1-CD147 complex interaction is more sensitive to mutations in the signature motif compared to the MCT1-GP70 complex, suggesting this region could explain the chaperone specificity between GP70 and CD147.

## Discussion

The N-terminal signature motif is one of two highly conserved motifs in the MCT family (14). These motifs facilitate the homodimerization of MCT2 (18), but have otherwise not been shown to play a functional role. Here, we show the N-terminal motif is sensitive to mutation and natural variation in a manner that can disrupt MCT1-chaperone interactions. The signature motif mutations that most significantly impacted the MCT1-CD147 interaction, G19C and W20A, reduced complex stability, plasma membrane expression, and subsequent cellular substrate uptake (Table 2). The defects in trafficking were not consistent between the two chaperones tested, CD147 and GP70, because W20A was not affected when co-expressed with GP70, suggesting possible different stabilization mechanisms. In contrast, mutations of nearby D15 and W18 play a minor role in disrupting chaperone function. Control mutations, N187A-MCT1 and F367Y-MCT1, are known for impacting MCT1-CD147 dimerization (19) and the MCT1 active site (25), respectively. The loss of CD147-mediated trafficking of G19C-MCT1 and W20A-MCT1 was comparable to N187A-MCT1, and the loss of substrate transport was comparable to F367Y-MCT1. The loss of proper localization due to signature motif mutations underscores its crucial role in MCT1-chaperone association.

**Table 2:** Summary table of the effects of MCT1 mutations on protein function and stability

MCT1 mutant	Location	MCT1 internalization	Bromopyruvate resistance	Complex thermostability	Monomer thermostability
D15A	N-terminus	↑	No Change	↓↓↓↓	↓
W18A	TM1	No Change	No Change	No Change	No Change
G19C^a^	TM1	↑↑	↑↑↑↑	↓↓↓↓	No Change
W20A	TM1	↑↑↑	↑↑↑↑	↓	No Change
N187A	TM6	↑↑↑↑	↑↑	No Change	↑
F367Y	TM10	No Change	↑↑↑↑	No Change	↑↑↑↑

a Observed in Thymic carcinoma (GDC UUID: fa780e9e-ac3a-5d71-9b01-54282d303a88).

Although much of the interaction between the signature motif and the CD147 CTD was not modeled in recent cryo-EM structures (19, 20), the Alphafold2 models shown here (Fig. 5) and cellular FRET assays (32) support the proximity of the MCT1 N-terminus and the CD147 C-terminus. Prior deletion studies further suggest that the C-terminal region of CD147 (residues 235–269) serves as an essential site for complex maturation (33). Our CD147 truncations validate this result and highlight the importance of residues 235 to 254 for protein-protein interaction (Fig. 1*D*). Despite the significant truncations made to CD147-Δ235, the protein still traffics to the plasma membrane, suggesting proper membrane insertion (Fig. S5). In contrast, the N-terminal CD147 Ig domains are dispensable for the copurification of MCT1 (8); they instead play a role in facilitating transport (34). The cryo-EM structure of MCT1-GP70 revealed an extracellular hydrogen bond between MCT1 and the GP70 Ig domain, which is not present in any MCT1-CD147 structures (19). Therefore, it is likely that CD147 relies on the involvement of its CTD to compensate for the lack of ectodomain-mediated interactions. Finally, because several of the signature motif residues and the CD147 CTD are in disordered regions, their interaction could generate multiple conformational states, or be “fuzzy” (35).

Although multiple mechanisms may explain how mutations not engaging in direct contact with CD147 or GP70 residues can destabilize the complexes, the structural models suggest indirect effects. *In silico* modeling of MCT1-CD147 using the cryo-EM structures shows the G19C mutation may induce an intramolecular clash with TM4 (Fig. 5*D*). Considering previous structural studies have shown that TM1 of MCT1/2 is highly mobile (18, 19), this clash may displace TM1, positioning it towards the C-terminus/transmembrane region of the chaperone and destabilizing the complex (Fig. 5*F*). W20 forms an intramolecular hydrogen bond between its indole nitrogen and the backbone carbonyl of G17, which is lost by W20A (Fig. 5*E*). The disruption of this critical architecture may interfere with the CD147 CTD interaction. Notably, the W20A mutation did not significantly destabilize the MCT1-GP70 complex, which reflects our prediction that the GP70 CTD does not closely interact with the MCT1 N-terminus (Fig. 5*F*). Since MCT1 monomeric stability is unaffected by G19C and W20A (Fig. 4), the signature motif mutants most likely disrupt a key region conferring specificity to the protein-protein interaction. Because previous structural studies have identified lipids involved in the stability of other heteromeric SLCs (36, 37), future research should examine the possibility of lipid-mediated interactions that affect MCT1 chaperone binding sites.

The signature motif G19C mutation was identified in a thymic cancer case through the Genomic Data Commons (GDC), a repository for clinical and genomic data linked to cancer patients (21). The current model of MCTs’ role in cancer describes an increased expression to facilitate the Warburg Effect and metabolic symbiosis (38, 39). However, thymic cancer tumor cells have a decreased cellular viability upon MCT1-mediated acetate uptake (40). In environments with high extracellular acetate, thymic cancer cells initiate apoptosis from high intracellular acetate concentrations and intracellular acidification (40, 41). This may provide selective pressure in the opposite direction to reduce MCT1-mediated transport activity to avoid the uptake of toxic substrates or an increase in intracellular acidification (29). Based on the available evidence, it is possible that an acquired G19C mutation could be protective against toxic substrate import by reducing MCT1-CD147 thermal stability and trafficking functions.

Overall, our results indicate that alterations to the N-terminal segment of MCT1 disrupt complexation with CD147, leading to a loss of complex stability, protein retention in the ER, and a decrease in MCT1-mediated transport. While these observations provide valuable insights, further structural elucidation of the MCT1-CD147 and MCT1-GP70 complexes will be crucial for a comprehensive understanding of the underlying molecular mechanisms that govern the MCT1-chaperone interaction.

## Experimental procedures

### Construct design and mutagenesis

Codon-optimized human MCT1 and GP70 were cloned into the pCDNA3.4 vector (Invitrogen) by Gibson assembly using recombinant gBlocks (IDT) (42). For MCT1, a template containing monomeric enhanced GFP (A206K) with a C-terminal polyhistidine tag (10×-His) was first constructed, and then a sequence of MCT1 with an N-terminal FLAG sequence (DYKDDDDK) was inserted at the N-terminus of GFP to create Flag-MCT1-GFP-10xHIS (referred to as MCT1-GFP hereafter). The full-length GP70 construct (Strep-GP70) contained the Twin-Strep tag (WSHPQFEKGGGSGGGSGGSAWSHPQFEK) at its C-terminus. The full-length, Δ254 (254–269 deletion) and Δ235 (235–269 deletion) constructs of human CD147 were cloned into the pcDNA3.4 vector using gene synthesis services (Twist). These sequences contained an internal HA-tag (YPYDVPDYA) inserted between residues 20 and 21, following the CD147 signal sequence. Site-directed mutagenesis was performed to introduce MCT1 mutations D15A, W18A, G19C, W20A, N187A, and F367Y using desalted oligonucleotides from Sigma Aldrich. All constructs were sequence verified prior to use (Elim Biopharmaceuticals).

### Cell lines and expression

Suspension Expi293F cells were cultured in Expi293 expression medium (Thermo Scientific, A14635) and maintained in a humidity-controlled incubator set to 37 °C with 8% CO_2_. Cells were transiently transfected with MCT1-GFP, HA-CD147, or Strep-GP70 constructs. Transfections were performed at a density of 2.5 million cells per milliliter with 1 μg of DNA per ml of cell culture using polyethyleneimine (PEI) (Polysciences, 24765-1) in a 1:1 (w/w) PEI to DNA ratio. Cells were supplemented with 8 mM sodium butyrate at 20 h post-transfection, and protein expression was allowed for 48 h before cell harvest for subsequent experiments.

### Pulldown assay on co-transfected constructs

Expi293F cells were transiently transfected with MCT1-GFP and CD147-FL, CD147-Δ254, or CD147-Δ235 using Expifectamine (Thermo Scientific, A14524) per manufacturer instructions and 1 μg of DNA per mL of cells. After 48 h, cells were collected by centrifugation (600*g*) and resuspended in lysis buffer (50 mM Tris pH 8.0, 150 mM NaCl, and APL protease cocktail consisting of 0.8 μM aprotinin, 0.2 μM pepstatin A, and 0.25 μM leupeptin hemisulfate) supplemented with 1.0% LMNG-CHS (10:1 ratio). Cells were lysed overnight at 4 °C with end-over-end mixing in lysis buffer, followed by centrifugation at 13,000 rpm for 10 min at 4 °C to remove insoluble fragments. The resulting whole-cell lysate was then incubated with G1 FLAG-affinity resin (GenScript, L00432) at a ratio of 40:1 (v/v) at 4 °C with end-over-end mixing overnight. Following three washes with 20 column volumes of lysis buffer supplemented with 0.05% LMNG:CHS (10:1) to remove unbound proteins, elution was performed by incubating the resin with 250 μg/ml of FLAG peptide resuspended in lysis buffer. Co-elution was evaluated by Western blot analysis using intrinsic GFP fluorescence (for MCT1 detection) and the anti-CD147 antibody (Cell Signaling Technologies, 13287S).

### Immunofluorescence

Transiently transfected Expi293F cells were immobilized on polylysine-L treated glass coverslips 48 h after transfection. Cells were then fixed with formaldehyde and permeabilized with 0.1% Triton X-100 in DPBS. Cells were next blocked with 1% BSA and incubated with anti-HA (Thermo, 26183) or anti-Strep (GenScript, A01732-100), at a 1:250 dilution in 0.1% BSA for 1 h at room temperature. After washing three times with DPBS to remove excess antibody, cells were incubated with Goat anti-Mouse IgG (H + L) Cross-Adsorbed Secondary Antibody, Alexa Fluor 568 (Invitrogen, A-11004) at a 1:500 dilution in 0.1% BSA for 1 h at room temperature. Cells were stained with antifade mounting media containing DAPI (Vector Laboratories, H-1800–2) and imaged on a Zeiss LSM880 fluorescence microscope with an Airyscan detector. Image analysis was performed using the multi-clock scan plugin (26) for ImageJ (NIH) software. Percent GFP and anti-HA/anti-Strep signal internalization were calculated for each cell using the Equation 1:

(1) $$\frac{AUC\;of\;signal\;0-70\%\;of\;cell\;radius}{AUC\;of\;signal\;from\;0-120\%\;of\;cell\;radius}=\%\;Signal\;Internalized$$

### Bromopyruvate viability assay

Expi293F cells were cultured and transfected as above. Cells treated with MCT1 inhibitor were given 250 nM AR-C155858 (Ambeed, A373794-1 mg) 24 h post transfection and were incubated for 10 min at 37 °C. 3-Bromopyruvate (Sigma, 16490-10G) was diluted in DPBS and added to cells 24 h post transfection. Cells were diluted 10-fold in DPBS immediately before the addition of 0.2 μM SYTOX Blue (Thermo, S34857). The diluted cells were incubated for at least 5 min before measuring the viability with the BD FACSCanto II Flow Cytometer. At least 5000 GFP-positive cells were counted per sample. Viability was normalized by Equation 2:

(2) $$\frac{\%\;Viability\;of\;treated\;cells}{\%\;Viability\;of\;untreated\;cells\;}\;\times \;100=\%\;Normalized\;Viability$$

The flow cytometry data were analyzed in the DIVA software and statistical analysis was performed in Prism GraphPad.

### GFP-based thermal shift assay

A crude membrane GFP-based thermal shift assay was performed as previously described (31). Expi293F cells were transfected and incubated as above. Crude membrane fractions were prepared from at least a biological duplicate of cells expressing MCT1 and CD147 variants. Cell pellets were resuspended in 50 mM Tris pH 8.0, 150 mM NaCl, and APL protease cocktail. Cells were lysed by eight rounds of sonication at 30% amplitude for 30 s followed by a 60 s incubation on ice. Crude membranes were harvested by ultracentrifugation and stored at −80 °C until needed.

Crude membranes were solubilized in 1.0% LMNG-CHS (10:1 ratio), 50 mM Tris pH 8.0, 150 mM NaCl, and APL protease inhibitor cocktail. The total protein of the solubilized crude membrane was quantified *via* Bradford assay (BioRad, 5000205) and normalized to 3.5 mg/ml of total protein. Then, a technical triplicate of the sample was aliquoted into PCR strips and heated at the desired temperature for 10 min in a BioRad thermocycler. Samples were then recovered on ice before being clarified at 5000*g*, 45 min, 4 °C. The supernatant fluorescence was measured at 488/512 nm using a Synergy Neo2 Multi-mode Microplate Reader (BioTek). The melting curves were fit with the Boltzmann Sigmoidal in Prism GraphPad Version 10.0.3 (217).

### Western blot analysis

Western blot analysis was performed on pulldown samples and samples collected at various temperature endpoints in the thermal shift assay. Samples were resolved on a 10% acrylamide SDS-PAGE gel. The polyacrylamide gel was transferred to a PVDF membrane (Millipore Sigma, IPVH00010) and blotted with 1:1000 dilution of anti-CD147 antibody (Cell Signaling Technologies, 13287S). The bands were visualized with horseradish peroxidase conjugate anti-rabbit antibodies (Cell Signaling Technologies, 7074P2) at a 1:2000 dilution and chemiluminescence detection using Clarity Western ECL Substrate (BioRad, 1705060). The MCT1 subunit was detected using intrinsic fluorescence with blue epi light excitation and a 530/28 nm filter.

### Structure prediction by AlphaFold2

AlphaFold2.3-multimer structural predictions were generated from MCT1 (Uniprot Identifier P53985), CD147 (Uniprot identifier P35613-2), and GP70 (Uniprot Identifier Q6PCB8) primary sequences. Models were generated using Alphafold2-multimer processed locally. The MMseqs2 multiple sequence alignment was used with a template date of November 20th, 2023. The FoldDock prediction (22) of MCT1-CD147 was retrieved from the API (/ProtVar/api/v3/api-docs) using the Uniprot identifiers from above.

### Statistical analysis

Statistical analysis was performed on quantitative data comparing each mutant to wild-type protein using Prism GraphPad Version 10.0.3 (217). An ordinary one-way ANOVA with Dunnett’s test for multiple comparisons was used to compare statistical significance, with nonsignificant (ns) data defined as *p* >0.05, and significant means defined as ∗*p* ≤0.05, ∗∗*p* ≤0.01, ∗∗∗*p* ≤0.001, and ∗∗∗∗*p* ≤0.0001. Signature motif conservation was visualized using the WebLogo server (43).

## Data availability

Data, AlphaFold models, and micrographs are available upon request.

## Supporting information

This article contains supporting information.

## Conflict of interest

The authors declare that they have no conflicts of interest with the contents of this article.
