# Supplementary material for: The N-terminal signature motif on the transporter MCT1 is critical for CD147-mediated trafficking
Source: J Biol Chem. 2024 May 22;300(6):107333. doi: 10.1016/j.jbc.2024.107333 (PMC11176948; doi:10.1016/j.jbc.2024.107333)
Supplement: Supporting Figures S1–S5 [file mmc1.pdf]

Supporting Information
Supplemental Figure 1

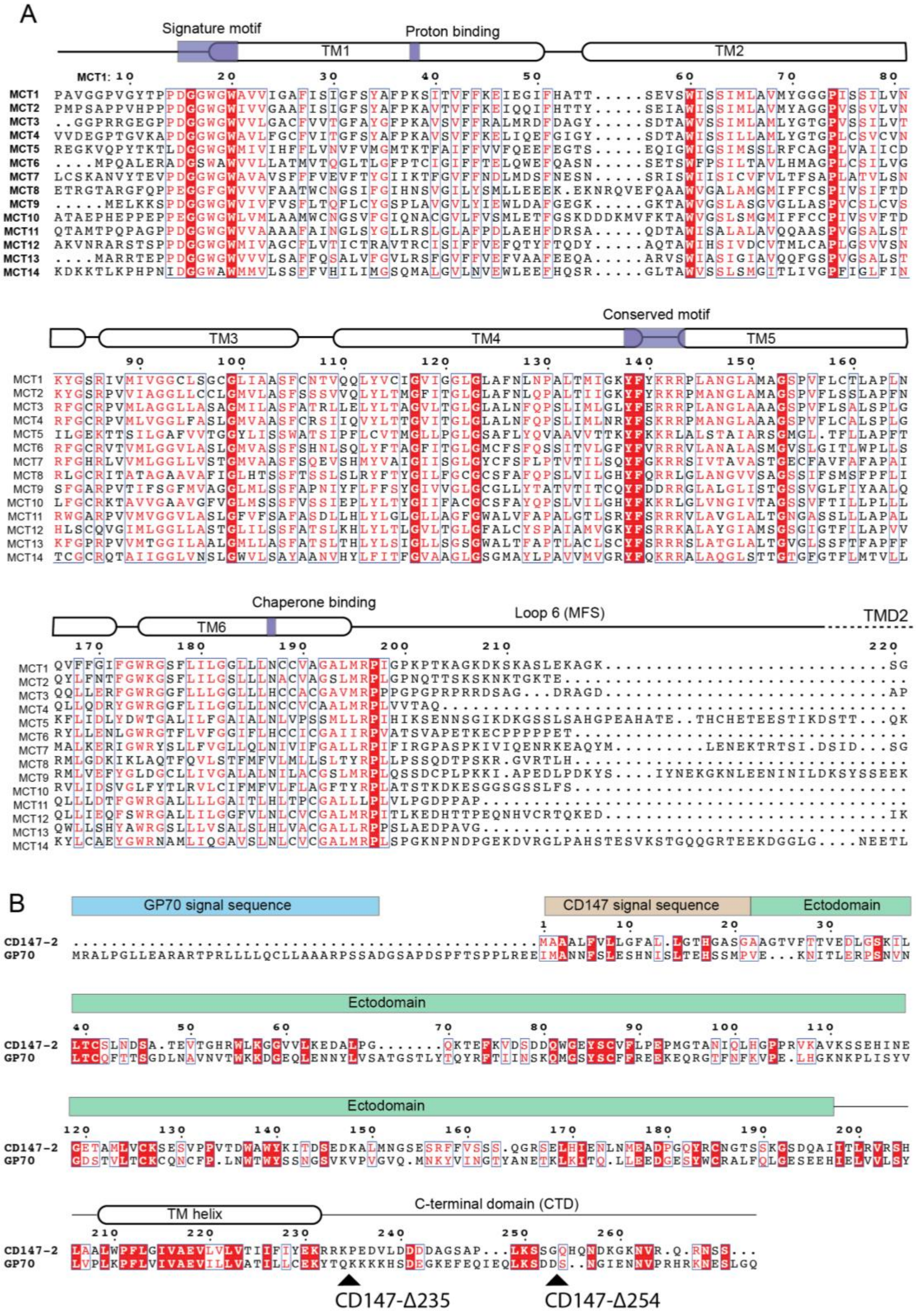

Figure S1. Sequence alignments of MCT1-14 and CD147/GP70 with notable features annotated. A) Sequence alignment of transmembrane domain 1 of MCT1-14 with transmembrane helices (TM) and motifs notated. B) Sequence alignment of CD147 and GP70 with structural elements labeled and truncation sites that were utilized in the MCT1 pulldown assay.

Supplemental Figure 2

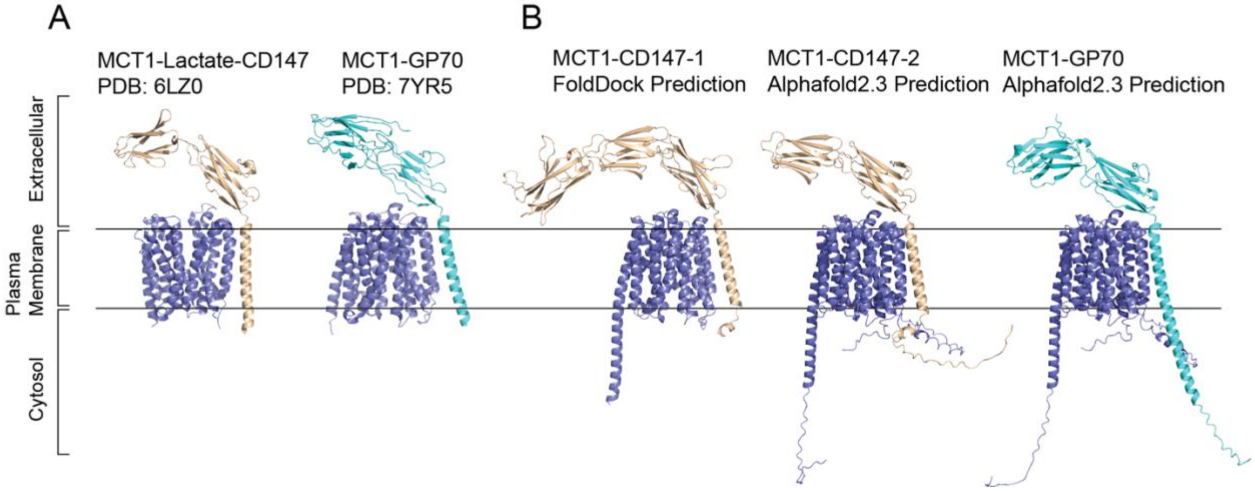

Figure S2. Experimental models do not resolve the cytosolic residues of MCT1, or the C-terminus of chaperone bound. A) Experimentally resolved structures of MCT1-CD147 (6LZ0) and MCT1-GP70 (7YR5). B) Protein prediction by FoldDock, and full-length predictions of MCT1-CD147 and MCT1-GP70 by Alphafold2.3-multimer.

Supplemental Figure 3

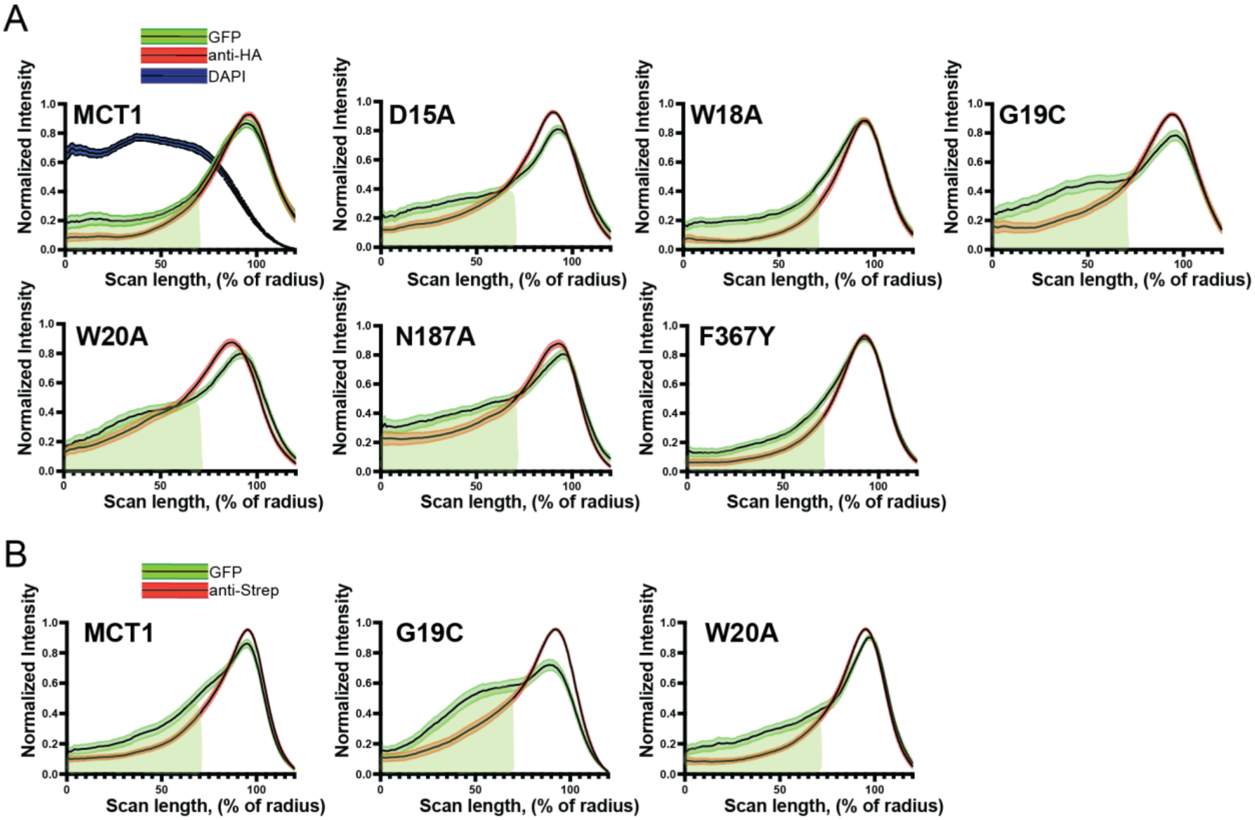

Figure S3. Clock scan profiles of MCT1-GFP signal and HA-CD147 or Strep-GP70 signals. A) Multi-clock scans of MCT1 variants with CD147 co-expression in HEK293F cells. Average intensity of each channel's signal shown with the area under the curve for 70% of the cell radius depicted in green shading. B) Multi-clock scan profiles for MCT1 variants co-expressed with Strep-GP70. Mean ± SEM, >45 cells from 3 biological replica.

Supplemental Figure 4

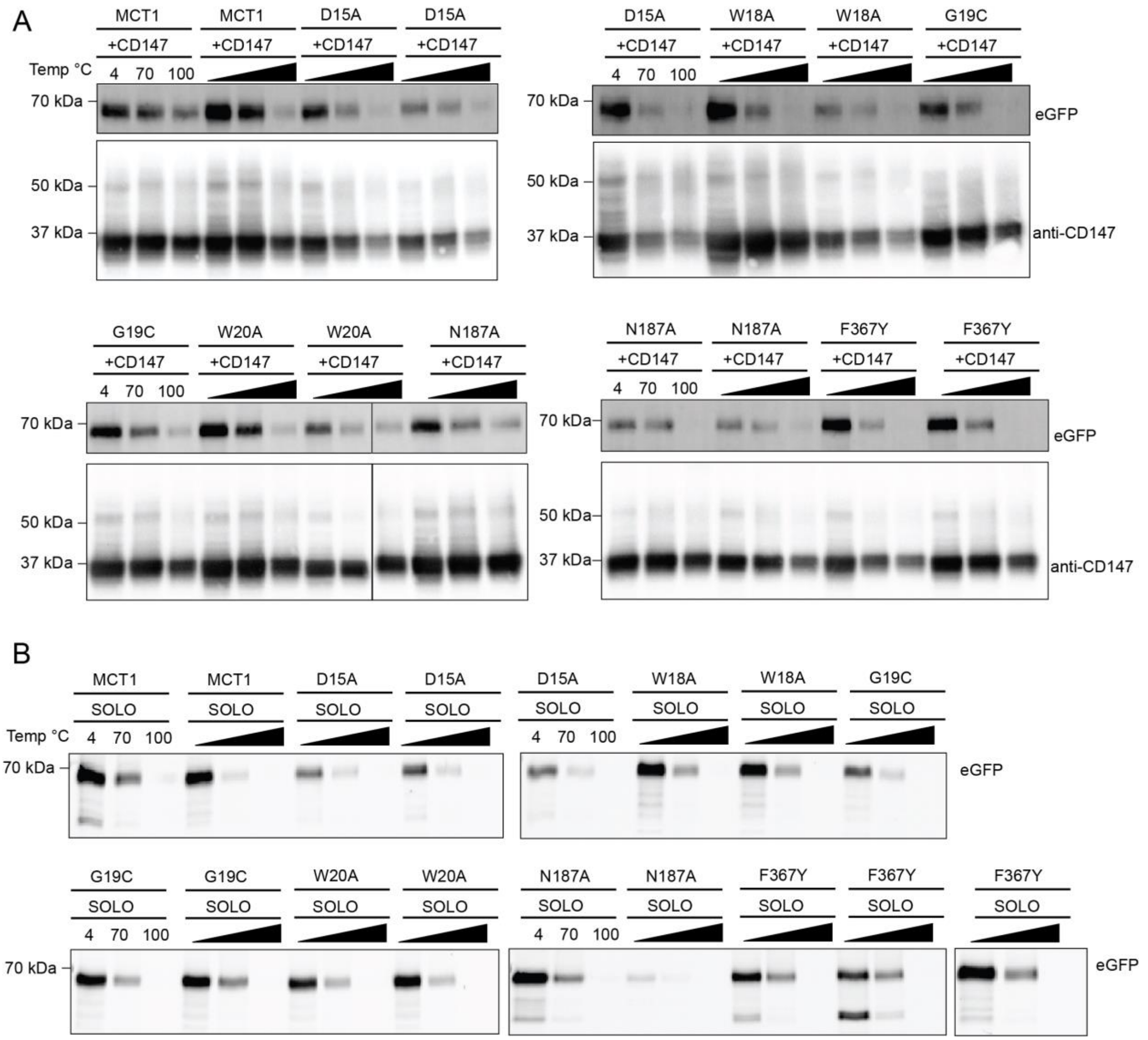

Figure S4. Western blot analysis and in-gel fluorescence of solubilized crude membrane subjected to temperature ramping. A) Western blot analysis of the MCT1-CD147 complex melting point from a temperature ramp of 4°C, 70°C, and 100°C endpoints. B) In-gel fluorescence of samples from MCT1-solo expressions from an endpoint temperature ramp.

Supplemental Figure 5

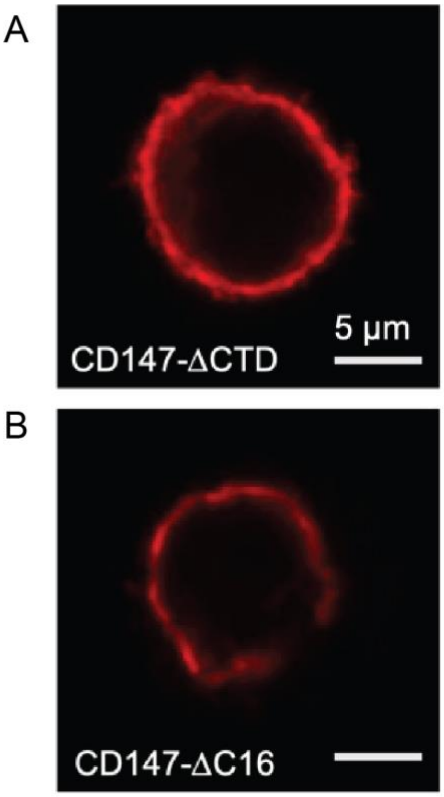

Figure S5. CD147 truncations localize to the plasma membrane in Expi293F cells. A) Cell transfected with CD147-ΔCTD (residues 1-235). B) Cell transfected with CD147-ΔC16 (residues 1-254).
